# 

**Published:** 1892-11

**Authors:** 


10 cts. a copy. NOVEMBER, 1892. $1.00 per year.

### HALL'S JOURNAL OF HEALTH

ESTABLISHED 1854

OFFICE, 206 BROADWAY.

Entered as Second-Class Matter at the Post-Office, New York.

